# When Can Antibiotic Treatments for Trachoma Be Discontinued? *Graduating* Communities in Three African Countries

**DOI:** 10.1371/journal.pntd.0000458

**Published:** 2009-06-16

**Authors:** Kathryn J. Ray, Thomas M. Lietman, Travis C. Porco, Jeremy D. Keenan, Robin L. Bailey, Anthony W. Solomon, Matthew J. Burton, Emma Harding-Esch, Martin J. Holland, David Mabey

**Affiliations:** 1 F.I. Proctor Foundation, University of California, San Francisco, California, United States of America; 2 Department of Ophthalmology, University of California, San Francisco, California, United States of America; 3 Department of Epidemiology & Biostatistics, University of California, San Francisco, California, United States of America; 4 Institute for Global Health, University of California, San Francisco, California, United States of America; 5 Department of Infectious and Tropical Diseases, London School of Hygiene and Tropical Medicine, London, United Kingdom; University of Cambridge, United Kingdom

## Abstract

**Background:**

Repeated mass azithromycin distributions are effective in controlling the ocular strains of chlamydia that cause trachoma. However, it is unclear when treatments can be discontinued. Investigators have proposed *graduating* communities when the prevalence of infection identified in children decreases below a threshold. While this can be tested empirically, results will not be available for years. Here we use a mathematical model to predict results with different graduation strategies in three African countries.

**Methods:**

A stochastic model of trachoma transmission was constructed, using the parameters with the maximum likelihood of obtaining results observed from studies in Tanzania (with 16% infection in children pre-treatment), The Gambia (9%), and Ethiopia (64%). The expected prevalence of infection at 3 years was obtained, given different thresholds for graduation and varying the characteristics of the diagnostic test.

**Results:**

The model projects that three annual treatments at 80% coverage would reduce the mean prevalence of infection to 0.03% in Tanzanian, 2.4% in Gambian, and 12.9% in the Ethiopian communities. If communities *graduate* when the prevalence of infection falls below 5%, then the mean prevalence at 3 years with the new strategy would be 0.3%, 3.9%, and 14.4%, respectively. Graduations reduced antibiotic usage by 63% in Tanzania, 56% in The Gambia, and 11% in Ethiopia.

**Conclusion:**

Models suggest that graduating communities from a program when the infection is reduced to 5% is a reasonable strategy and could reduce the amount of antibiotic distributed in some areas by more than 2-fold.

## Author Summary

Trachoma, the major cause of infectious blindness in the world, occurs when repeated infections of the ocular strains of *Chlamydia trachomatis* lead to a cascade of conjunctival scarring, in-turned eyelids and eyelashes, and eventually blindness due to corneal opacity. To reduce the prevalence of infection, the World Health Organization (WHO) advocates at least three annual community-wide distributions of oral antibiotics in affected areas. This approach has proven effective, but there is room to explore other treatment strategies which reduce the use of antibiotics. Here, we used mathematical models and data from three trachoma-endemic countries (Tanzania, The Gambia, and Ethiopia) to analyze different treatment strategies. In the simulations, we show that a *graduation* strategy can reduce antibiotic distributions more than 2-fold in moderately affected areas. Both treatment strategies provide favorable results in reducing the prevalence of ocular chlamydia, but high costs and the potential for resistance are important issues to consider when administering mass doses of antibiotics.

## Introduction

Over 40 million doses of oral azithromycin have already been distributed to control the ocular strains of chlamydia that cause trachoma [1]. The World Health Organization (WHO) advocates three annual community-wide distributions and continued treatment until clinical evidence of infection falls below a threshold where resulting blindness should not be a major public health care concern. These distributions have proven effective in reducing infection in longitudinal studies, individual-randomized trials, and community-randomized trials [2]–[14]. Studies have also suggested that in some circumstances distributions may be safely discontinued, either because infection has been eliminated, or the few remaining infections may disappear on their own [7], [8], [10], [12]–[14]. Laboratory testing offers the possibility of *graduating* communities that no longer need to receive mass antibiotics, reducing expense, side-effects, and the potential for developing drug resistance in other organisms such as *Streptococcus pneumonia* [13],[15]. Here we explore the effect of graduating communities from a treatment program, with *graduation* being defined as a point in time when a community will no longer receive mass antibiotic distributions because evidence of infection is below a prescribed threshold.

Less expensive diagnostic laboratory testing for infection may become available for trachoma programs in the near future. Estimates of the prevalence of infection using PCR-based tests can be made more cost-effective by sampling individuals within communities and by pooling several samples into one test [6],[10],[14],[16],[17]. There is hope that a low cost, point-of-care (POC) test will become available which will allow on-the-spot identification of infected communities [18]. Strategies to graduate communities when the prevalence of infection has decreased below a threshold are now being tested. However, the results will not be available for years, and only one strategy can be tested at a time. Here we use data from three African countries to fit a stochastic mathematical model, and then predict the results of two different treatment programs, three annual mass treatments consistent with WHO recommendations versus three annual mass treatments using strategies to graduate communities.

## Methods

### Clinical data

Data were collected in three countries at baseline, and 2 and 6 months after treatment as previously described [6],[8],[19]. Informed consent along with institutional review board approval was given in each of the studies as described [6],[8],[19]. Although there were differences in the underlying study designs in the three countries, the similarities allow comparable data sets to be abstracted from each. In all three sites, there was a baseline mass oral azithromycin distribution, and then no subsequent azithromycin treatment until after the 6 month collection. In all three sites, all individuals aged one year and older were offered a single dose of oral azithromycin during mass treatments. Pregnant women and those allergic to macrolides were offered alternative treatment (topical tetracycline). The right upper conjunctiva of each child was swabbed, and the swab was then tested for the presence of chlamydial DNA using Amplicor PCR (Roche Diagnostics, Branchburg, NJ). In Ethiopia, sixteen villages in the Gurage region of southern Ethiopia were randomly selected and 1–5 year old children, the ages most likely to harbor infection, were monitored [6]. In Tanzania and in The Gambia, swabs were taken from all individuals of all ages, although for the purposes of this study, we use only swabs taken from children aged 1–10 years. In Tanzania and The Gambia, 15 balozis (a household administrative unit consisting of about 10 households), and 14 villages, respectively, were followed [7],[8].

### Mathematical models

Previously, we constructed a simple stochastic SIS model (*S*usceptible, *I*nfected, *S*usceptible) of ocular chlamydial infection in a core group of children [20],[21]. In this report, the previous model was modified to include infection from outside of the community, and a transmission term that can vary between communities in the same region as a normally distributed random effect (to account for the known variability of communities). Treatment strategies that allowed graduation of communities when observed infection fell below a certain threshold, as detected by a POC test, were incorporated. Specifically, we constructed a Markov model by letting 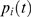 denote the probability that there are *i* infected individuals in the population at time *t* (where *i* varies from 0 to *N*). At scheduled treatments, we assumed that each infected individual had an 80% chance of being treated (the WHO-recommended coverage rate), and if treated would revert to being susceptible. Between periodic mass treatments, the model is a standard continuous time Markov process. We assumed equilibrium at baseline, that infected individuals recover naturally at rate *γ*, that uninfected individuals become infected at rate *β I/N* from sources within the community (with *β* = *R_0_*·γ), and at a rate of *ν* from outside the community (with *ν* decreasing to zero once wide-spread programs have begun), leading to the following set of *N+1* Kolmogorov-forward equations:
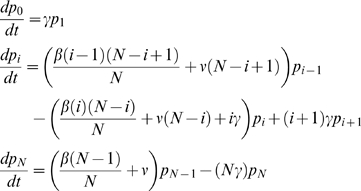



For clarity, we expressed *β* in terms of the basic reproductive number, R_0_ (where *β* = *R_0_*·γ). Note that R_0_ is defined as the mean number of secondary infectious cases caused by a single infectious case in an otherwise completely susceptible community [22]. At the time of the scheduled periodic mass treatments, we assume that each infected individual has a probability *c* of being treated (the effective coverage), with the number of infections post treatment being drawn from the corresponding binomial distribution.

Parameters for this stochastic model were fitted to baseline and 6-month data for each country using maximum likelihood estimation. We initiated simulations at the average prevalence for that region, and simulated the Kolmogorov-forward equations for 40 years to allow the distribution of prevalence to approximate the pre-treatment distribution at time point zero. We also initiated the model at the observed 2-month prevalence and simulated the equations for 4 months to estimate the expected distribution of prevalence at 6-months. The total log-likelihood was the sum of the baseline and the 6 month log-likelihoods for each of the communities in the area. Note that any event that occurred between baseline and 2-months (such as treatment, or mass re-infection from travel) would not bias these results [8]. Based on these Kolmogorov equations, the values of the parameters *R_0_*, standard deviation of *R_0_* (thus treating *R_0_* as a random effect), *γ*, and *ν* that maximized the probability of obtaining the observed baseline and 6-month data for that country (i.e. the likelihood) were determined using an iterative, hill-climbing algorithm. Numerical optimizations were repeated a minimum of 4 times from random starting points (because of the possibility any single run could converge to a local, rather than the global, maximum); each iteration converged to the same value. Furthermore, a grid search did not reveal any greater maxima.

The variance of these estimates was assessed by inverting the Hessian of the log-likelihood evaluated at the maximum likelihood estimate (although note that the 95% confidence interval could not include *ν = 0*, because in each country, a community went from 0 infections at 2 months to >0 infections at 6 months). Coverage was assumed to be 80%, and the average population size was set at the mean of empirical results from the surveyed communities in that region (Table 1). For sensitivity analyses, we ran 1000 simulations with the fitted parameters under different scenarios. We varied the sensitivity and specificity of the POC test, as well as the threshold for declaring graduation, keeping *R_0_*, standard deviation of *R_0_*, *γ*, and *ν* at the optima found for that region. If not being varied, the sensitivity and specificity of a POC diagnostic test were set at 70% and 99% respectively, and the prevalence threshold for graduating communities of ≤5%. All analyses were carried out in *Mathematica* 5.2.

**Table 1:** Characteristics of three data sets.

Country	Mean Baseline Prevalence (95% CI)	Mean number of Children per Village (Ages 0–9)	Total Number of Communities	References
**Tanzania**	16% (10%, 22%)	24	15	19
**Gambia**	10% (0%, 20%)	38	14	8
**Ethiopia**	64% (56%, 72%)	50	16	6

## Results

Characteristics of the observed data from the three countries are shown in Table 1. Tanzania had a higher mean prevalence than The Gambia, but there was a larger variation in prevalence in Gambian communities, with some having as high as 40% and nearly two-thirds not having any infection identified. Ethiopia had high prevalences in all communities. These areas of Tanzania and The Gambia would be considered meso-endemic or hypo-endemic areas, and the area of Ethiopia hyper-endemic. Predicted model parameters are displayed in Table 2. The mean basic reproduction number, *R_0_*, and the estimate of the standard deviation of *R_0_* (treating the particular *R_0_* in a community as a random effect), the recovery rate, *γ*, and the exogenous infection rate, *ν*, are displayed for the data sets from each country, along with the 95% confidence intervals for these parameter estimates. The Ethiopian data resulted in the highest estimated mean *R_0_*, while the largest coefficient of variation for *R_0_* was found in The Gambia, consistent with the observed heterogeneity between villages found there. The exogenous infection rate is dependent not only on the overall prevalence of infection in an area, but the proximity of the defined communities in each country.

**Table 2:** Estimated model parameters for each of the three data sets.

Country	R_0_ (95% CI)	Standard Deviation of R_0_ (95% CI)	Recovery Rate, Gamma (95% CI) weeks^−1^	Exogenous infection rate, Nu (95% CI) weeks^−1^
**Tanzania**	0.89 (0.50, 1.27)	0.001 (0.000, 0.576)	0.037 (0.001, 0.073)	0.002 (0.000, 0.004)
**The Gambia**	1.01 (0.66, 1.36)	0.724 (0.001, 1.447)	0.052 (0.000, 0.113)	0.0002 (0.000, 0.001)
**Ethiopia**	3.14 (2.51, 3.77)	0.7315 (0.232, 1.231)	0.0123 (0.005, 0.02)	0.001 (0.000, 0.002)

**Figure 1:**
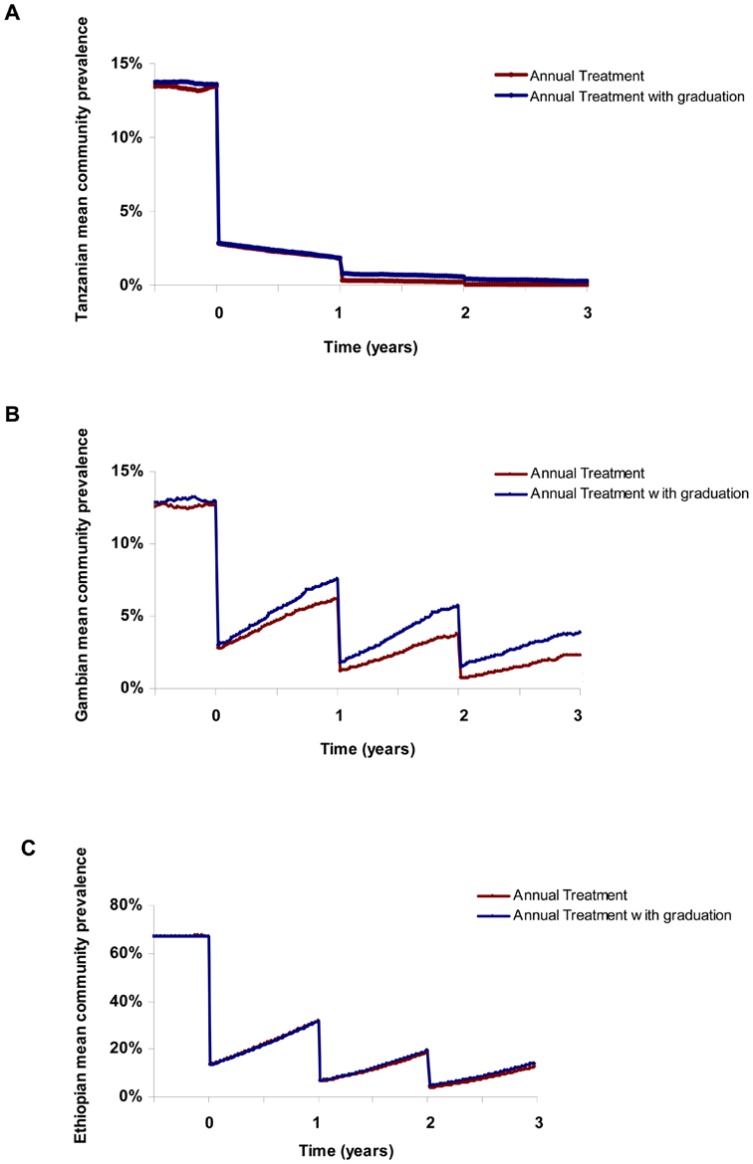
Simulations assessing the effect of graduation on prevalence of infection. Each curve represents the mean of 1000 stochastic simulations of community prevalence of ocular chlamydial infection in children with three annual mass treatments versus annual mass treatments with graduation, in Tanzania (A), the Gambia (B), and Ethiopia (C). In the graduation strategy, communities received an initial mass treatment, and two subsequent annual mass treatments until the prevalence was reduced below 5%.

**Figure 2:**
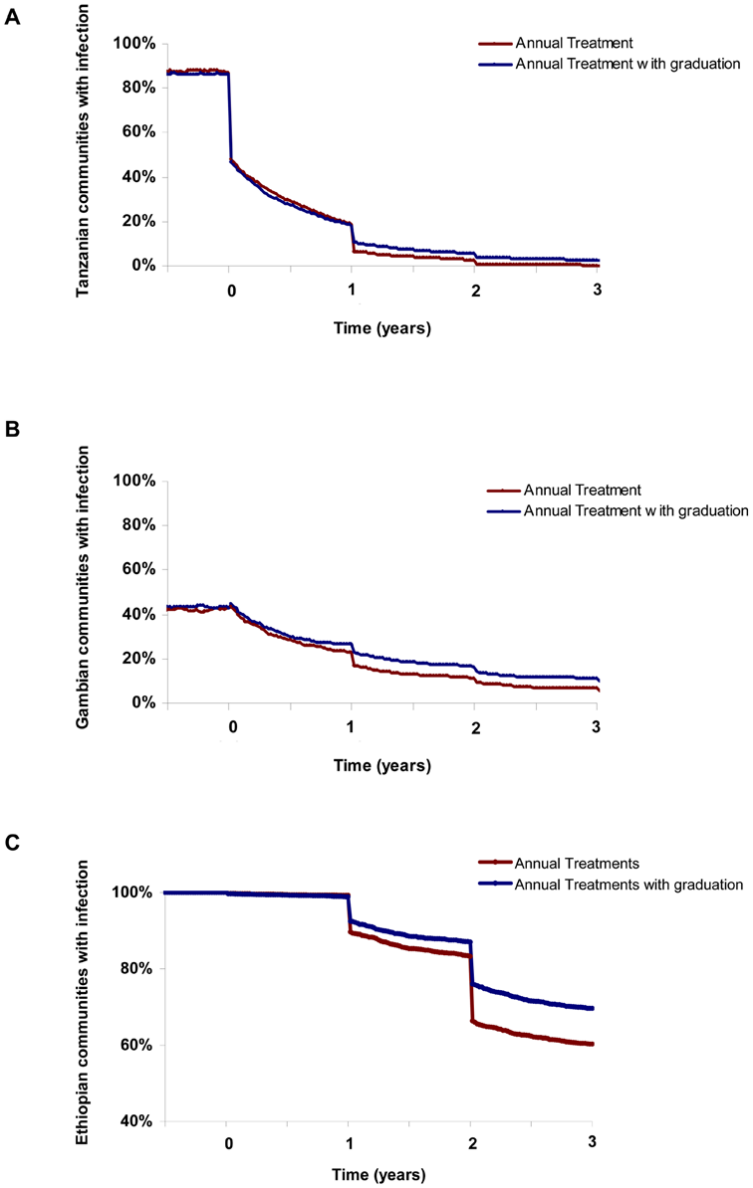
Simulations comparing the effect of graduation on eliminating infection in communities. Each curve represents the proportion of 1000 simulated communities which still harbor infection, in Tanzania (A), the Gambia (B), and Ethiopia (C). Note that in Tanzanian and Gambian simulations, some communities had no identifiable infection pre-treatment, which is consistent with the observed data).

The WHO-recommended strategy of three annual mass treatments (before subsequent re-evaluation for further treatment) resulted in an estimated mean prevalence of 0.0% in Tanzanian, 2.4% in Gambian, and 12.9% in Ethiopian communities at three years (Figure 1A, 1B, and 1C). In addition, results showed complete elimination of infection in 99.8% of Tanzanian communities, 93.3% of Gambian communities, and 39.8% of Ethiopian communities (Figure 2A, 2B, and 2C).

**Figure 3:**
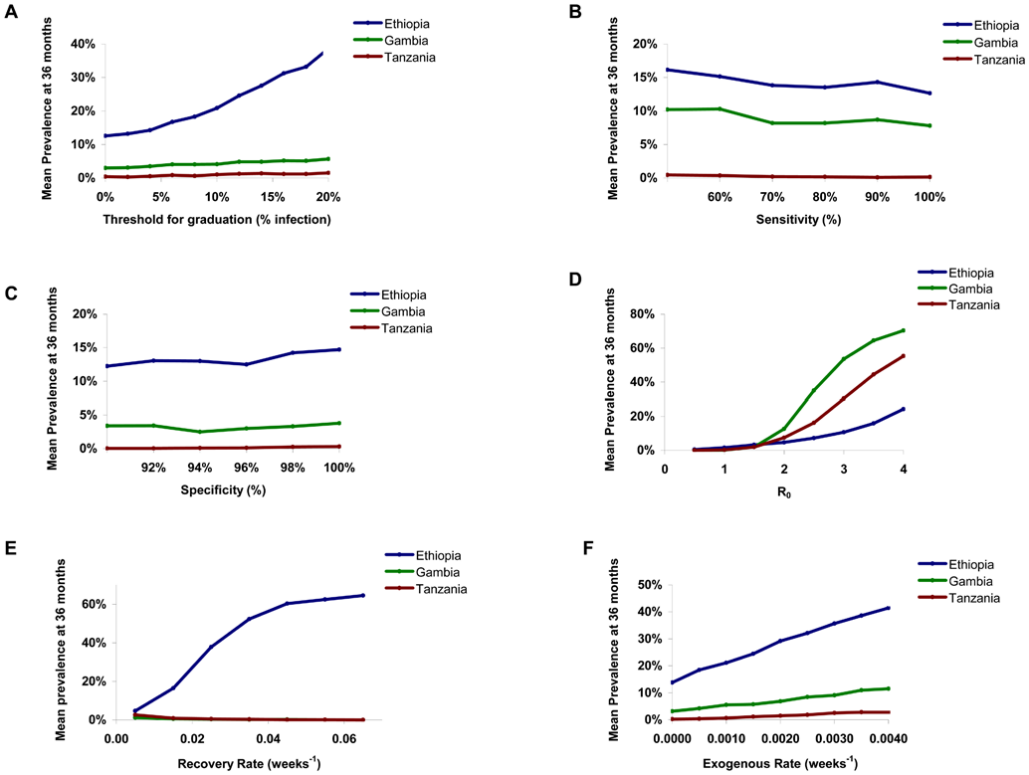
Sensitivity of simulated prevalence on the choice of parameter values. Here we in turn varied the threshold for graduation (A), sensitivity of the diagnostic test (B), specificity of the diagnostic test (C), R_0_ (D), recovery rate γ (E), and exogenous rate ν (F). The mean prevalence at 3 years for 1000 simulations is displayed as the parameter is varied, for each of the three countries. Parameters which were not varied were assumed to be as in Table 2.

**Figure 4:**
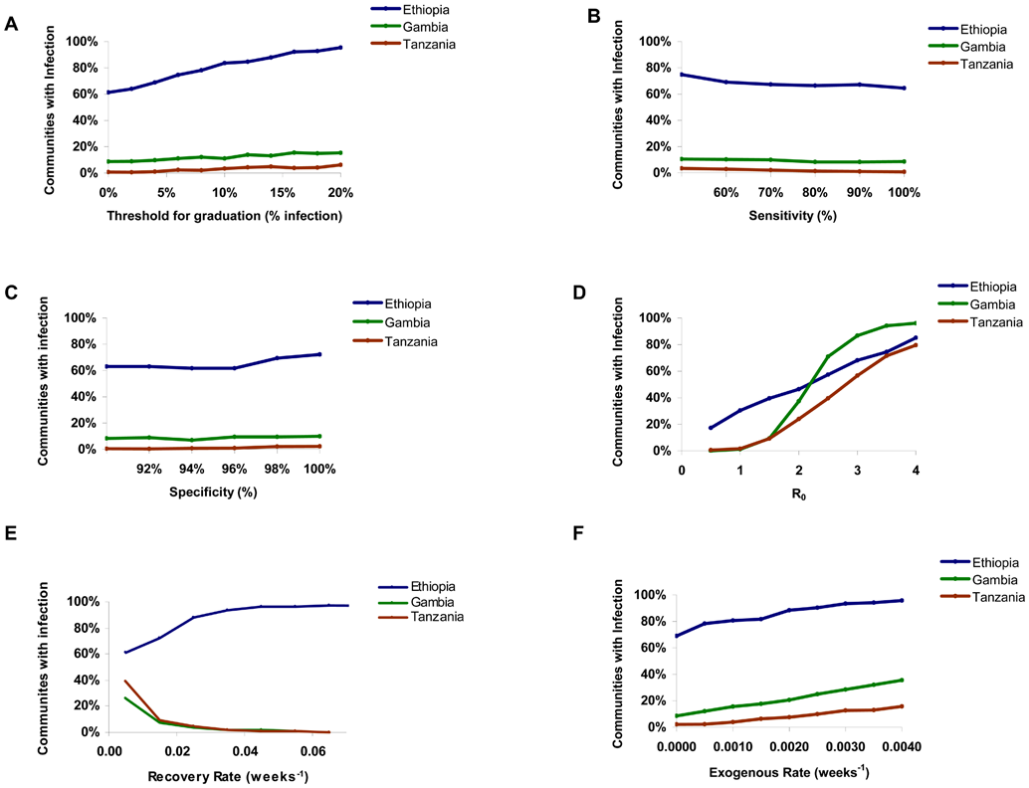
Sensitivity of elimination of infection on the choice of parameter values. The proportion of communities which still harbor infection at 36 months is displayed as the parameter is varied, for each of the three countries. Parameters varied are threshold for graduation (A), sensitivity of the diagnostic test (B), specificity of the diagnostic test (C), R_0_ (D), recovery rate γ (E), and exogenous rate ν (F). Parameters which were not varied were assumed to be as in Table 2.

With a strategy of graduating communities from further mass antibiotics when the observed prevalence of infection falls below 5%, the estimated mean prevalence at 3 years was 0.3% in Tanzania, 3.9% in The Gambia, and 14.4% in Ethiopia (Figure 1A, 1B, and 1C). The proportion of communities where elimination would be expected was 97.6% in Tanzania, 88.8% in The Gambia, and 30.0% in Ethiopia (Figure 2A, 2B, and 2C). Elimination in each of the three countries is affected by the threshold for graduation, sensitivity and specificity of the diagnostic test, *R_0_*, recovery rate, and the exogenous rate (Figures 3 and 4). Increasing the threshold for graduation marginally increased the median prevalence of infection at 36 months (Figure 3) and the proportion of communities in which infection could still be identified (Figure 4). As one might expect, increasing either *R_0_* or the exogenous rate increases prevalence and decreases the proportion of villages which attain infection elimination (Figures 3 and 4). However, these sensitivity analyses suggest that the simulation results are not dependent on the exact choice of the sensitivity and specificity of the diagnostic test. Increasing the recovery rate (γ) allows the prevalence of infection to return to the equilibrium of the region more rapidly.

**Figure 5:**
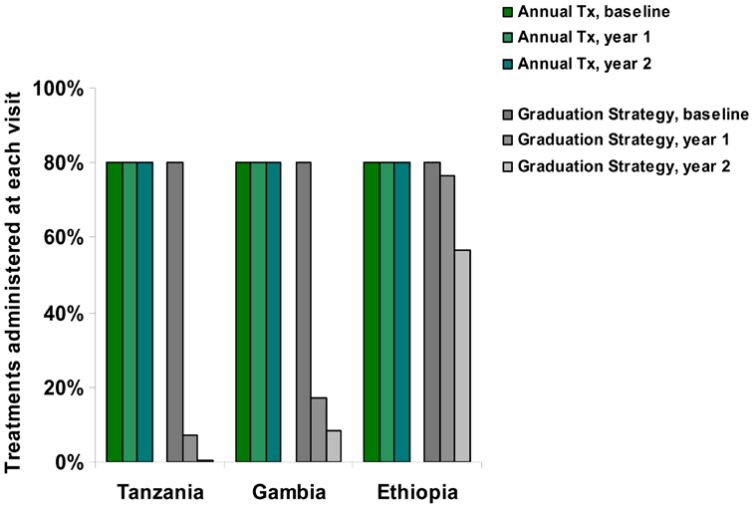
Treatment savings with graduation strategy. The percentage of individuals treated at each annual visit with mass treatment versus a graduation strategy. Note that we assume an 80% coverage for mass antibiotic treatments.

**Figure 6:**
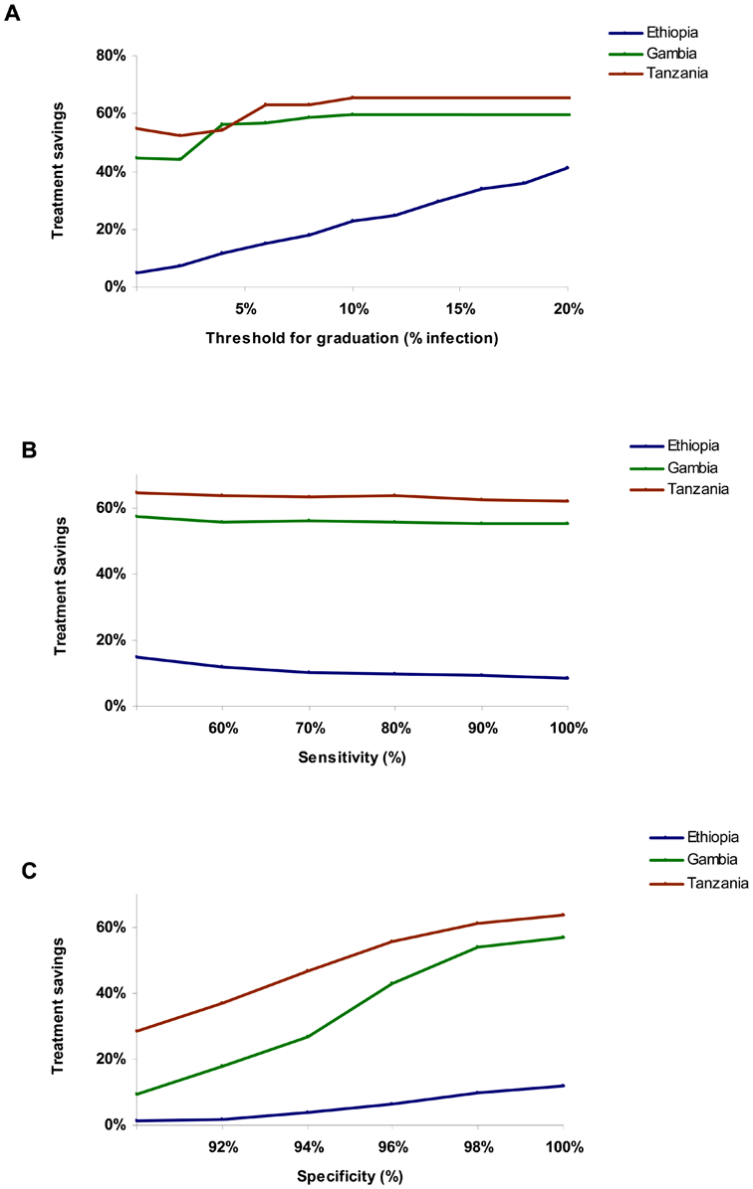
Sensitivity of treatment savings on parameter values. The percentage of treatments saved with a graduation strategy is displayed as the threshold for graduation (A), sensitivity of diagnostic test (B), or specificity of diagnostic test (C) is varied. Parameters which were not varied were assumed to be as in Table 2.

Figure 5 shows the amount of antibiotics used during three years of the graduation strategy, compared to three years of annual treatment. The graduation strategy, over the three year period, would reduce treatments by 63% in the Tanzanian communities, 56% in Gambian communities, and 11% in the Ethiopian communities, compared to annual treatments. These reductions in treatments are also affected by alterations in the threshold for graduation, test sensitivity, and test specificity (Figure 6). Increasing the specificity has a marked reduction in antibiotic use (Figure 6C). Note that if the specificity falls low enough, then more than 5% infections will be “observed” due to false positives. In this case, there will be no graduations, regardless of the true prevalence of infection.

## Discussion

The WHO's recommendation of a minimum of three annual antibiotic distributions with at least 80% coverage should work well in most areas. In the models representing Gambian and Tanzanian communities, infection was eliminated in more than 95% of communities during the first three years of a WHO-recommended treatment program, consistent with later observed results in the Tanzanian communities [13]. Note that the WHO recommends reassessment at this point, and further antibiotic distributions if the clinical signs of infection are still present. Most communities in these areas will have eliminated infection in children after 3 annual treatments.

In hyper-endemic areas like the studied in Ethiopia, we expect 3 years of treatment to eliminate infection in 40% of communities. This is consistent with a recent community-randomized clinical trial in a nearby area of Ethiopia [14]. We have previously suggested that biannual treatment in hyper-endemic areas may be a more successful strategy to eliminate infection, at least in a 2–5 year time frame [14],[23].

A strategy that graduates communities when the observed prevalence of infection in children falls below 5% appears to be reasonable. The models presented here predict that treatment could be discontinued in the vast majority of communities similar to those studied in Tanzania and The Gambia. Graduating communities had only a minimal effect on the mean prevalence of infection found in the area at the end of a 3-year trachoma program. This is because trachoma transmission is low in many of the Tanzanian and Gambian communities, and because the graduation strategy favors re-treating the communities which have higher transmission. We predict that antibiotic use could be reduced 2 to 3-fold by introducing such measures, and even more if the graduation threshold were applied before the first treatment—here, we treated all communities at least once.

The goal of trachoma programs is to efficiently use resources to eliminate trachoma as a public health concern. The cost of providing treatment for a community in Africa, estimated at $0.25–1.00 per individual, includes the costs of antibiotics, as well as wages for health workers and administrators [24]. These costs can be expected to result in a 75–90% reduction in ocular chlamydial prevalence at one year after treatment, per mass distribution [7],[9],[10],[14]. However, the cost-effectiveness of a program is determined by more than treatment costs and reduction of infection. Trachoma programs must decide which communities to treat, and when to stop treatment. As shown in this study, this decision will require knowledge of the prevalence of chlamydial infection, since areas with highly prevalent chlamydial infection will likely require longer periods of treatment. The costs of diagnosing ocular chlamydial infection vary. Currently, chlamydial infection is usually assessed with the clinical conjunctival exam [25]. Although the clinical exam is relatively inexpensive (costs include only the examiner's time), it is not particularly accurate in identifying chlamydial infection, particularly after treatment [26]. Chlamydial PCR is an alternative, though it is expensive when performed for an individual ($12–20 per test, personal communication, V. Cevallos, F.I. Proctor Foundation). Because trachoma programs base treatment decisions not on individuals, but on the entire community, samples could be pooled for PCR testing, which would result in considerable savings ($2–3 per individual tested, assuming 5 samples per pool) [16],[17]. A point of care test for chlamydia may also provide a less expensive method for diagnosing ocular chlamydia ($0.70 per individual tested) [18]. Besides the inclusion of these additional costs, there are additional outcomes that play a role in the cost-effectiveness of trachoma control. Specifically, while the outcomes of antibiotic treatment programs can easily be measured as the reduction in ocular chlamydial infection, the true outcome of interest is the reduction of blindness. However, the progression from infection to blindness in individuals can take decades, and rates are difficult to assess. Of note, treatment efforts besides antibiotics, such as promotion of face-washing and construction of latrines, may also be administered by trachoma programs, though the effectiveness of these measures has not been proven, making their role in cost-effectiveness difficult to study at present.

Mathematical models of trachoma control can be useful in determining optimal distribution strategies, and predicting what is expected from specific programs. Stochastic models have an advantage over deterministic models in this setting, because they include the effect of chance seen with the low levels of infection in communities near elimination. Models must also include variation between regions, since strategies that have proven effective in hypo-endemic areas may not translate to hyper-endemic areas. Models should also include variation between communities in the same region. To incorporate this community variation into a realistic model, we included a community-level random effect in transmission (specifically, variation in the underlying *R_0_* for each community). The results confirm that there is likely some variability between neighboring communities even beyond that which would be expected by chance. Incorporating further mixing structure, for example preferential mixing within a household, might create more variance between simulated communities, reducing the need to introduce a random effect at the community-level as we have done here. The graduation strategy appears to be effective over a wide range of parameter choices in the Tanzanian and the Gambian communities studied here. More endemic areas such as those found in Ethiopia, appear more sensitive to parameter choices, and are far more vulnerable to reinfection.

There is a long history of the use of mathematical models of the transmission of infectious diseases. These are often used to make theoretical points, which do not require precise parameter estimates. Even when parameters are fitted to data, they are rarely fitted to data from more than one community. Community trials in trachoma control have provided longitudinal prevalence data in 14–16 communities in each of three sub-Saharan African countries. There are still many limitations of this simple model which can be explored in the future. We have included only transmission in children, the age group known to harbor most of the infection, but adults could be included with available data. We have assumed that individuals do not gain or lose immunity over the 3 year program, and that there is no antibiotic resistance; the importance of these factors has been debated [11],[27],[28]. Our model defines the rate of contact to be independent of the population size (since the mass action term is divided by the effective population size *N*)—other assumptions such as density dependent transmission are also possible. Finally, we have assumed that treatment of children on a given visit is independent of past treatment history. Were treatments to miss the same 20% of children each time, then infection might linger longer in this subgroup.

Repeated mass oral azithromycin distribution will be effective in reducing the prevalence of ocular chlamydia. But distribution costs are high, and side effects and the potential for resistance are important issues. Graduating communities when diagnostic testing reveals a prevalence of ocular chlamydia of 5% or less appears to be an appropriate strategy in most areas. More frequent treatment and different stopping rules may be required for more hyper-endemic areas similar to the region of Ethiopia studied here.
